# Investigation of fluconazole susceptibility to *Candida albicans* by MALDI-TOF MS and real-time PCR for *CDR1, CDR2, MDR1* and *ERG11*

**DOI:** 10.1186/s12866-022-02564-4

**Published:** 2022-06-10

**Authors:** Chanika Maenchantrarath, Pradchama Khumdee, Seksun Samosornsuk, Narissara Mungkornkaew, Worada Samosornsuk

**Affiliations:** 1 Graduate Program in Biomedical Sciences, Faculty of Allied Health Sciences, Thammasat University, Pathumthani Province Bangkok, Thailand; 2 Microbiology Laboratory Unit, Department of Central Laboratory and Blood Bank, Faculty of Medicine Vajira Hospital, Navamindradhiraj University, Bangkok, Thailand; 3 Graduate Program in Medical Technology, Faculty of Allied Health Sciences, Thammasat University, Pathumthani Province Bangkok, Thailand; 4 Department of Medical Technology, Faculty of Allied Health Sciences, Thammasat University, Rangsit Campus, Pathumthani, Thailand; 5 Microbiology Laboratory Unit, Thammasat University Hospital, Pathumthani Province Bangkok, Thailand

**Keywords:** *Candida albicans*, MALDI-TOF MS, Fluconazole susceptibility testing, *CDR1*, *CDR2*

## Abstract

**Background:**

*C. albicans* is a pathogenic yeast that is the most common cause of fungal infections in humans. Unfortunately, the yeast’s resistance to the antifungal medication fluconazole (FLC) is increasing; furthermore, testing its susceptibility to FLC by conventional methods takes time, resulting in treatment failure. The susceptibility of *C. albicans* to FLC was investigated using MALDI-TOF Mass Spectrometry and Real-time PCR tests for *CDR1, CDR2, MDR1* and *ERG11*. Overall, 32 *C. albicans* strains made up of four reference strains (three FLC susceptible [S] and one FLC resistant [R], one spontaneous mutant strain [FLC susceptible-dose-dependent (SDD)] and 27 clinical strains obtained from two Thai University Hospitals) were tested for susceptibility to FLC. The following tests were performed: SensititreYeastOne and broth microdilution method, FLC resistant expression mechanism by Real-time PCR, and the major peak determination by MALDI-TOF MS.

**Results:**

The change of *CDR1* and *CDR2* mRNA expression was only significantly observed in SDD and R strains. MALDI-TOF MS was performed after incubation for six hours; the change of mass spectral intensity at range 3376–3382 m/z (major peak) was significantly related to FLC susceptibility as SDD (decreased at 4 µg/mL and increased at 8 µg/mL), S (all increased), and R (all slightly decreased or no change). All 27 clinical strains showed FLC minimum inhibitory concentrations (MIC range 0.25-2 µg/mL), no change in *CDR1* and *CDR2* expression and S major peak type. The FLC resistant *C. albicans* with *CDR1*and *CDR2* expression may possibly affect the change of mass spectral intensity at range 3376–3382 m/z.

**Conclusions:**

The MALDI-TOF MS may be used to simultaneously classify and predict FLC resistant *C. albicans* strains associated with *CDR1* and *CDR2* expression. Further studies are essential to clarify the methodology and improve the reliability of this assay for routine diagnosis.

## Background

The incidence in hospitals of fungal diseases, especially candidemia, is increasing the risk of morbidity and mortality [1]. *Candida albicans* is the most common reported species, followed by *C. glabrata*, *C. tropicalis, C. parapsilosis* and *C. krusei* (*Pichia kudriavzevii*) [2]. Fluconazole (FLC), a triazole antifungal medicine, is the most widely used treatment for this infection. Fluconazole has a fungistatic effect by inhibiting cytochrome P450 enzyme lanosterol demethylase (14α-demethylase), encoded by *ERG11*, in the ergosterol biosynthesis pathway. Many *C. albicans* clinical isolates overexpress *ERG11*, the gene encoding the target of the azoles. However, in many cases the level of overexpression is minimal or else observed in combination with other resistance mutations, making it difficult to assess the direct impact of such overexpression on the resistant phenotype. In addition to *ERG11* overexpression, two main classes of efflux proteins -- ATP-binding cassette (ABC) transporters encoded by *CDR1* and *CDR2*, and Major facilitator superfamily (MFS) encoded by *MDR1–c*an increase resistance to FLC by reducing the effective intracellular drug concentration [3, 4]. The azole resistance associated with *ERG11*, *MDR* or *CDR* overexpression has been reported in many cases [5, 6]. Currently, the broth microdilution (BMD) method is the gold standard of antifungal susceptibility testing (AFST) of *Candida* spp. This method of determining the MIC level of antifungal agents has been standardized by the Clinical and Laboratory Standards Institute (CLSI) and European Committee on Antibiotic Susceptibility Testing (EUCAST). However, this method is time-consuming and tedious, and an alternative method is desirable. The matrix assisted laser desorption ionization-time of flight mass spectrometry (MALDI-TOF MS) is generally used for microbial identification by referring to validated databases of reference spectra [1]. Several studies have adapted the MALDI-TOF MS for antifungal susceptibility testing of bacteria and fungi because of its simple, rapid, high-throughput, low waste, and low expense method [7–9]. The aim of this study is to investigate FLC susceptibility of *C. albicans* using MALDI-TOF MS and Real-time PCR for *CDR1, CDR2, MDR1* and *ERG11*.

## Results

### MIC level of control strains, clinical strains and spontaneous mutant strains

Of the FLC susceptibility testing with four reference strains, *C. albicans* MYA2876, *C. albicans* ATCC90028 and *C. albicans* MYA4440 were susceptible (S) to FLC at MIC level of 0.25, 0.25 and 0.5 µg/mL, respectively, whereas *C. albicans* ATCC96901 was resistant to FLC at MIC level of ≥ 64 µg/mL. With up to 10 consecutive serial passages of spontaneous mutations, ΔMYA4440 -- the mutation strain of *C. albicans* MYA4440 -- was susceptible-dose dependent to FLC (MIC = 4 µg/mL), while G950 strain did not change the MIC level. All 27 clinical strains of *C. albicans* were susceptible to FLC at MIC range 0.5-2.0 µg/mL (Table 1). Twelve strains collected from Thammasat University Hospital using SensititreYeastOne (SYO) were susceptible to fluconazole, voriconazole, anidulafungin, caspofungin and micafungin when applying CLSI CBPs. When applying the epidemiological cutoff values (ECVs), all the isolates had wild type phenotype drug susceptibility to amphotericin, flucytosine, posaconazole and itraconazole (Table 2).

**Table 1:** MIC levels of all *Candida albicans* by broth microdilution method

Sample code	FLC-MIC (µg/ml)	Classification	Group
MYA2876	0.25	S	Susceptible
ATCC90028	0.25	S	Susceptible
MYA4440	0.5	S	Susceptible
ΔMYA4440	4	SDD	Susceptible-dose dependent
ATCC96901	≥ 64	R	Resistant
Y4	0.25	S	Susceptible
Y5	0.25	S	Susceptible
Y22	0.25	S	Susceptible
Y59	0.5	S	Susceptible
Y62	0.5	S	Susceptible
Y63	0.25	S	Susceptible
Y77	0.25	S	Susceptible
Y83	0.25	S	Susceptible
Y93	1	S	Susceptible
Y101	0.5	S	Susceptible
Y102	0.5	S	Susceptible
Y121	0.5	S	Susceptible
S100	0.25	S	Susceptible
R380	0.25	S	Susceptible
S13	0.25	S	Susceptible
S16	0.25	S	Susceptible
G853	0.25	S	Susceptible
G189	0.25	S	Susceptible
G950	2	S	Susceptible
H282	0.25	S	Susceptible
G627	0.25	S	Susceptible
G1002	0.25	S	Susceptible
H2053	0.25	S	Susceptible
R237	0.5	S	Susceptible
S261	0.25	S	Susceptible
G582	0.25	S	Susceptible
S95	0.25	S	Susceptible

*MIC* Minimum inhibitory concentration, *FLC* Fluconazole, *S* Susceptible, *SDD* Susceptible-dose dependent, *R* Resistant

**Table 2:** MIC levels of 12 *Candida albicans* strains from Thammasat Hospital by Sensititre YeastOne

Sample No.	FLC		AND		AB		MF		CAS		5-FC		PZ		VOR		IZ	
	MIC	Interpretation	MIC	Interpretation	MIC	ECV Interpretation	MIC	Interpretation	MIC	Interpretation	MIC	ECV Interpretation	MIC	ECV Interpretation	MIC	Interpretation	MIC	ECV Interpretation
Y4	0.25	S	0.12	S	0.5	wt	0.015	S	0.25	S	0.25	wt	0.03	wt	0.008	S	0.12	wt
Y5	0.25	S	0.12	S	0.5	wt	0.015	S	0.25	S	0.25	wt	0.03	wt	0.008	S	0.12	wt
Y22	0.25	S	0.12	S	0.5	wt	0.015	S	0.03	S	0.06	wt	0.03	wt	0.008	S	0.06	wt
Y59	0.5	S	0.06	S	0.25	wt	0.008	S	0.03	S	0.12	wt	0.03	wt	0.008	S	0.06	wt
Y62	0.5	S	0.06	S	0.25	wt	0.008	S	0.03	S	0.06	wt	0.03	wt	0.008	S	0.06	wt
Y63	0.25	S	0.015	S	0.25	wt	0.01	S	0.03	S	0.06	wt	0.03	wt	0.008	S	0.03	wt
Y77	0.25	S	0.015	S	0.5	wt	0.008	S	0.03	S	0.12	wt	0.03	wt	0.008	S	0.03	wt
Y83	0.25	S	0.015	S	0.25	wt	0.008	S	0.03	S	0.12	wt	0.03	wt	0.008	S	0.03	wt
Y93	1	S	0.06	S	0.25	wt	0.008	S	0.03	S	0.12	wt	0.06	wt	0.015	S	0.12	wt
Y101	0.5	S	0.015	S	0.25	wt	0.015	S	0.03	S	0.06	wt	0.03	wt	0.008	S	0.06	wt
Y102	0.5	S	0.015	S	0.25	wt	0.008	S	0.015	S	0.06	wt	0.008	wt	0.015	S	0.03	wt
Y121	0.5	S	0.15	S	0.25	wt	0.008	S	0.03	S	0.12	wt	0.015	wt	0.008	S	0.03	wt

*FLC* fluconazole, *AND* anidulafungin, *AB* amphotericin B, *MF* micafungin, *CAS* caspofungin, *5-FC* flucytosine, *PZ* posaconazole, *VOR* voriconazole, *IZ* itraconazole, *ECV* Epidemiological cutoff value, *wt* wildtype

### *CDR* efflux pump overexpression

**Fig. 1:**
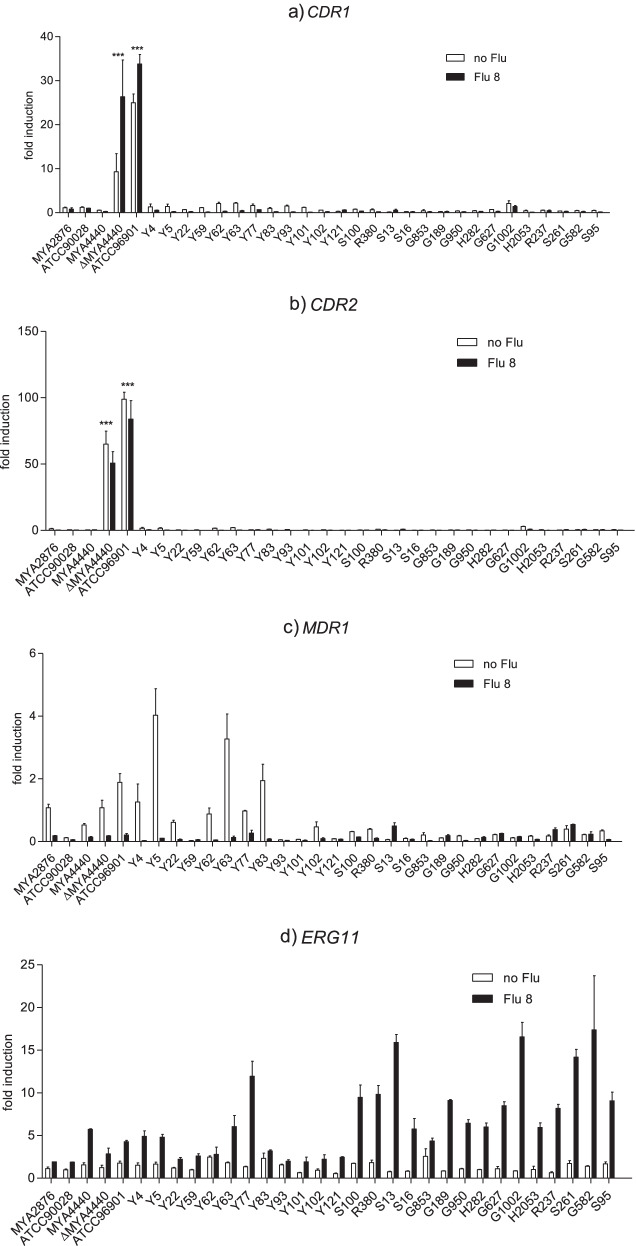
The mRNA expression of target genes (**a**) *CDR1* (**b**) *CDR2* (**c**) *MDR1* and (**d**) *ERG11* in *C. albicans* susceptible strain (*n* = 30; MYA2876, ATCC90028, MYA4440, and 27 clinical strains), fluconazole susceptible-dose dependent strain (*n* = 1; ΔMYA4440) and resistant strain (*n* = 1; ATCC96901), comparison between without fluconazole condition and after fluconazole 8 µg/ml addition for 24 hours

Figure 1a and b indicate *CDR* efflux pump overexpression in *C. albicans* strains: the SDD (ΔMYA4440) and R (ATCC96901) strains significantly overexpressed both *CDR1* and *CDR2* (*p *-value < 0.0001) compared to S strains. Compared to *C. albicans* MYA4440 (wild type) strain (MIC 0.5 µg/mL), its mutant strain ΔMYA4440 (MIC 4 µg/mL) expressed *CDR1* and *CDR2* at a higher level. After incubation with 8 µg/mL of FLC for 24 h, the SDD strain (ΔMYA4440) and R strain (ATCC96901) significantly overexpressed *CDR1* (*p* -value < 0.0001) but significantly repressed *CDR2* (*p *-value < 0.0001), while S strains (MYA2876, ATCC90028 and MYA4440) and all clinical S strains didn’t significantly express *CDR1* and *CDR2* (*p* -value > 0.0001).

### *MDR1* efflux pump overexpression

*MDR1* expression was not significantly different among S (MYA2876), SDD (ΔMYA4440) and R (ATCC96901) strains (*p* -value > 0.0001) (Fig. 1c). Without the addition of FLC, some SDD, R and some S strains -- including three S reference strains and some S clinical strains -- expressed *MDR1*. After incubation with 8 µg/mL of FLC for 24 h, most strains were not significantly changed in *MDR1* expression. *MDR1* overexpression was observed in some strains (Y59, S13, G189, H282, G627, G1002, R237, S261 and G582). Nonetheless, except 3 strains (Y4, Y5 and Y63) showed significant result as *MDR1* repression (*p* -value < 0.0001).

### *ERG11* target enzyme overexpression

Figure 1d shows *ERG11* overexpression. No statistically significant differences of *ERG11* expression (*p* -value > 0.0001) were seen among all strains. After incubation with 8 µg/mL of FLC for 24 h, all strains showed increased *ERG11* expression. The 15 strains of clinical susceptible strains (55.56%) showed significantly increased *ERG11* expression (*p* -value < 0.0001), while 12 strains (44.44%) were not significantly different to the control strains (*p* -value > 0.0001).

### The protein spectrum related to the fluconazole resistance of *C. albicans* in control standard strains

**Fig. 2:**
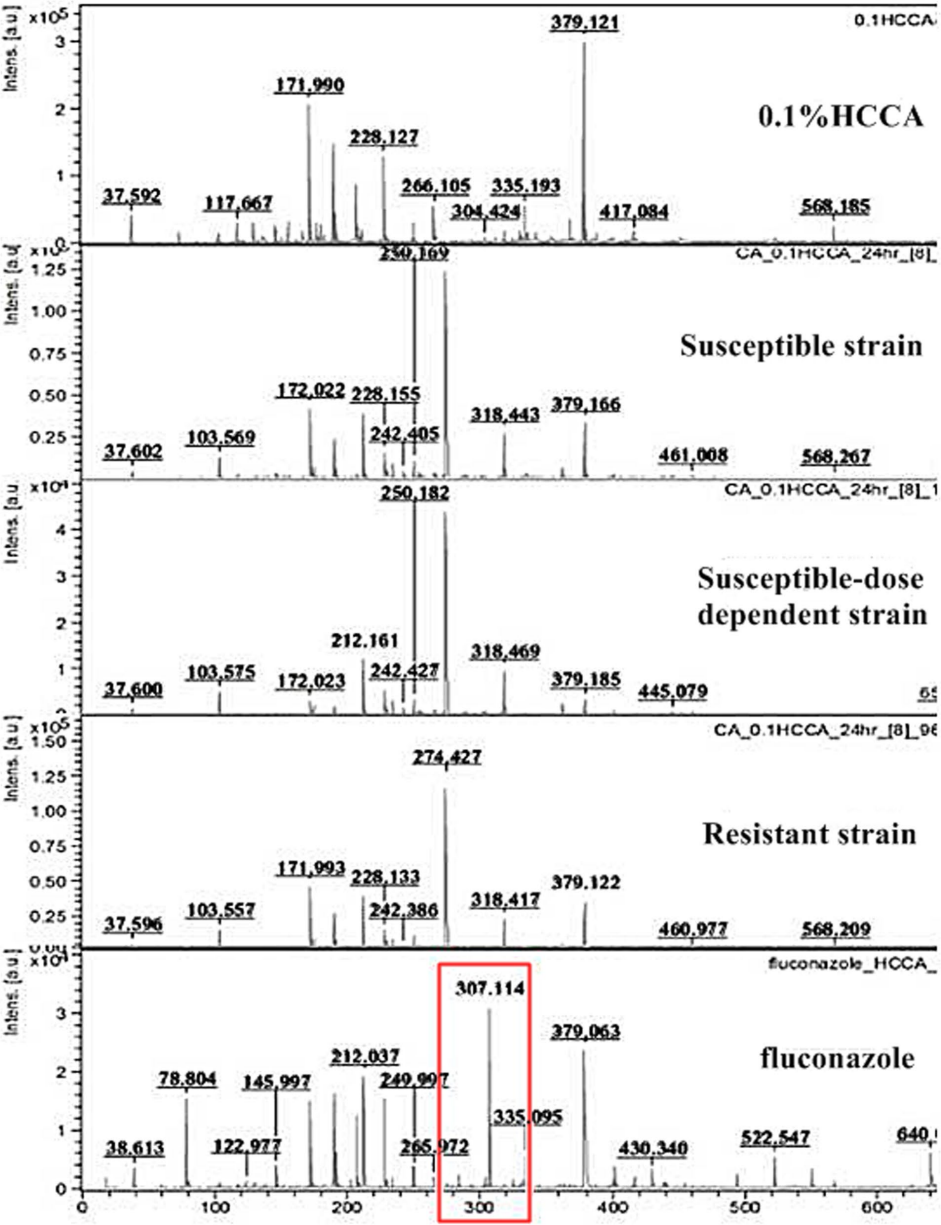
The spectrum of control 0.1% HCCA, fluconazole susceptible, susceptible-dose dependent strain, resistant *C. albicans* strains, and fluconazole position at 307 m/z (red box) (from top to bottom) using the reflector mode

**Fig. 3:**
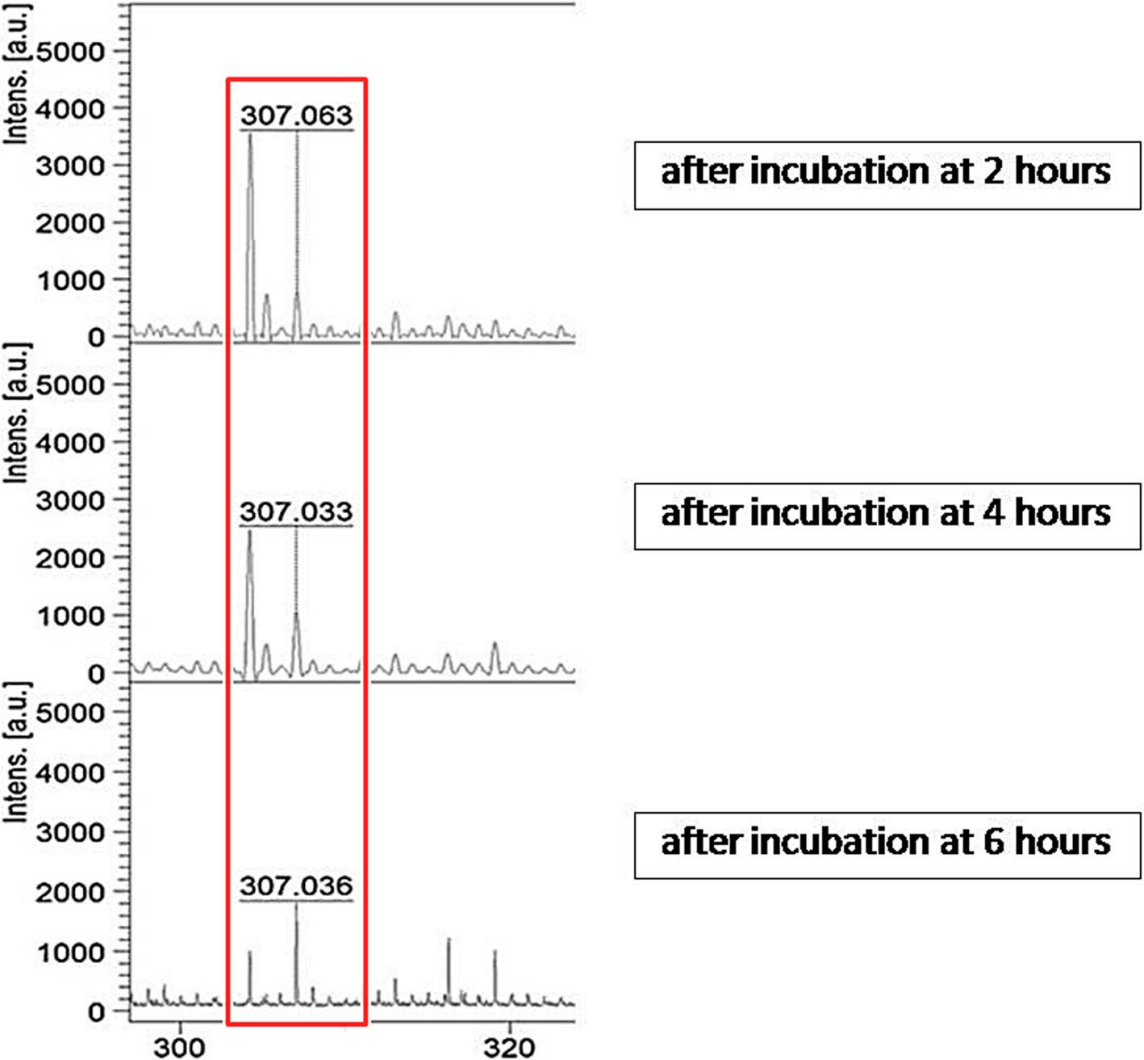
The spectrum of fluconazole position at 307 m/z (red box) after incubation at 2, 4 and 6 h (from top to bottom) using the reflector mode

**Fig. 4:**
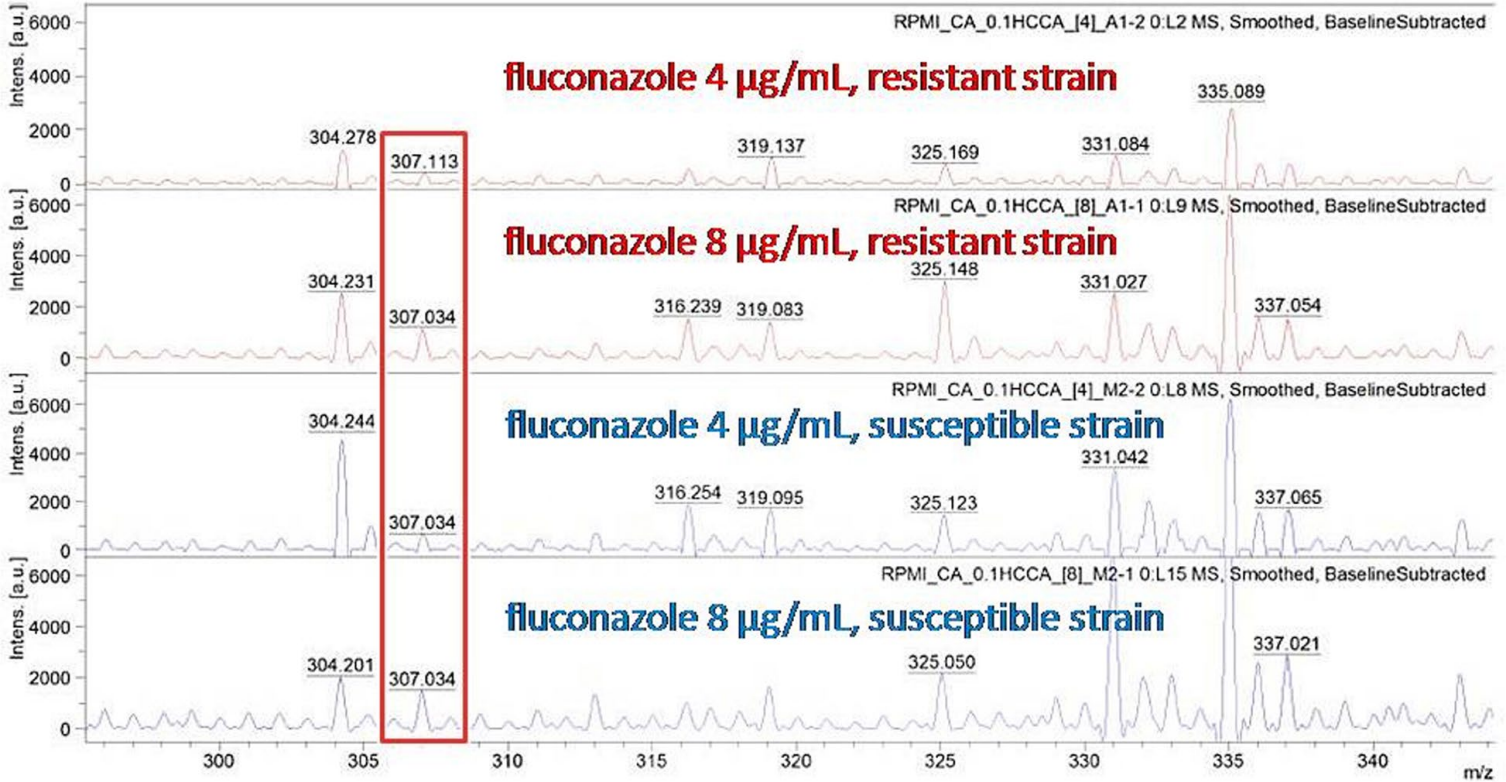
The comparison of fluconazole spectrum, position at 307 m/z (red box), after incubation at fluconazole concentration 4 and 8 µg/ml of resistance strain and susceptible strain, respectively (from top to bottom)

This experiment was to observe FLC mass spectral change among S, SDD and R control strains after incubation with 4 and 8 µg/mL for 6 h. Before initiating the experiment, the quality control of reagent, microorganisms, drug, and incubation time period was investigated as shown in Figs. 2 and 3. Figure 2 shows the mass spectral range 0-600 m/z of 0.1%HCCA, FLC S *C. albicans* strain, FLC SDD *C. albicans* strain, FLC R *C. albicans* strain and FLC. The mass spectral intensity of FLC appeared at position 307 m/z in the spectrum, while those of 0.1%HCCA and FLC S, SDD and R strains did not show a corresponding peak at 307 m/z. Testing after FLC incubation for 2, 4 and 6 h, the mass spectral intensity of FLC was consistently present (Fig. 3). This indicates that the incubation period does not affect the FLC mass spectrum. Next, S and R *C. albicans* strains were treated with 4 and 8 µg/mL of FLC and incubated for 6 h. Testing then showed the mass spectral intensity of 8 µg/mL FLC addition was higher than that of 4 µg/mL FLC addition. No FLC hydrolysis was detected in this experiment (Fig. 4). A peak at spectrum position 307 m/z can found in every condition of fluconazole addition, although the intensity at 8 µg/mL of fluconazole concentration will be higher than at 4 µg/mL of fluconazole concentration (Fig. 4). This suggests that FLC hydrolysis is not the mechanism of the action of FLC in *C. albicans*. Therefore, we cannot use FLC mass spectrum position to determine among FLC S, SDD and R strains.

**Fig. 5:**
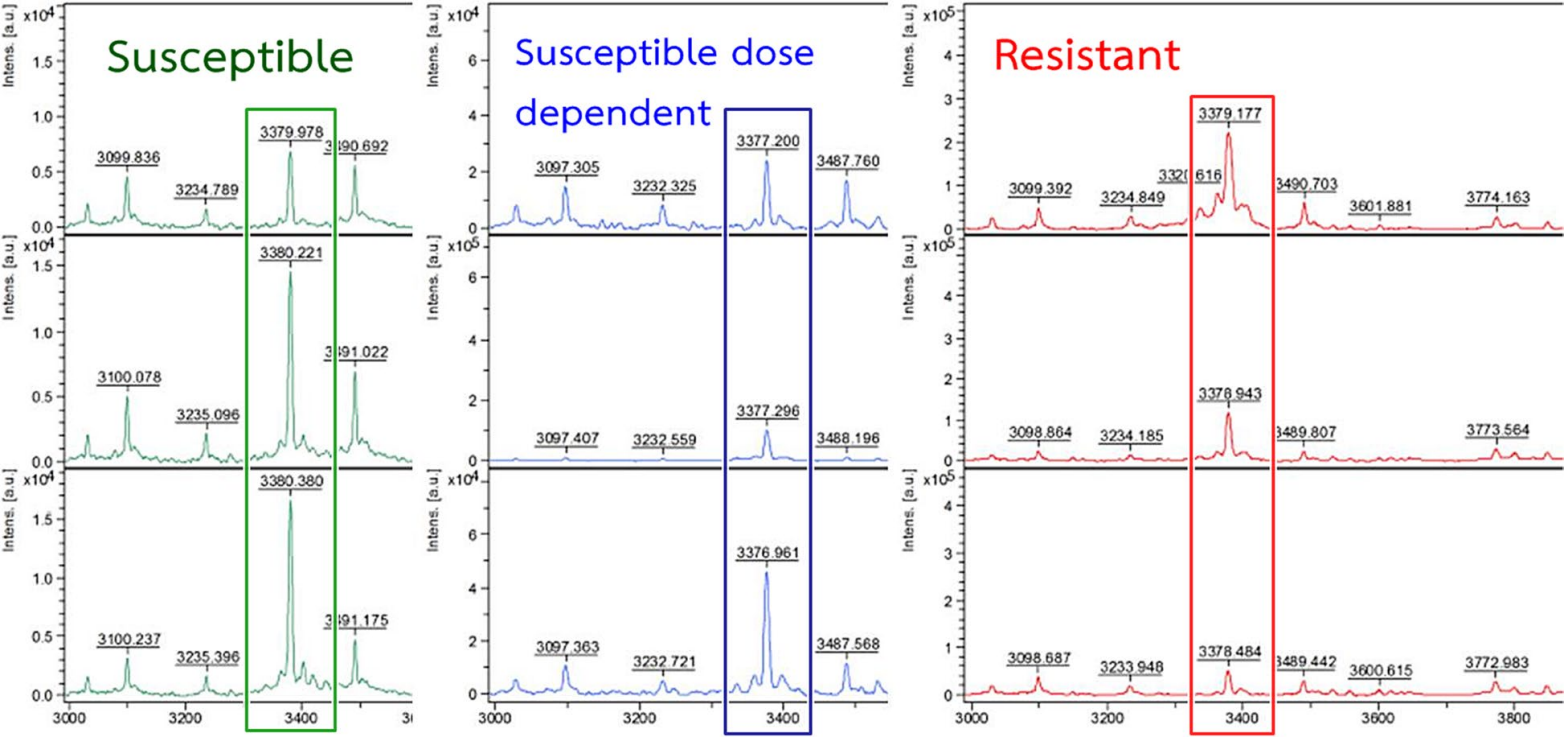
The spectrum intensity change at position 3376–3382 m/z of fluconazole-susceptible *C. albicans* (green box) fluconazole-susceptible-dose dependent *C. albicans* (blue box) and fluconazole-resistant *C. albicans* (red box) after incubation with fluconazole concentration 0 µg/mL (top row), 4 µg/mL (middle row) and 8 µg/mL (bottom row) for 6 hours

Then, the spectra of *C. albicans* at mass spectral range 2,000–20,000 m/z were investigated after incubation with 0 (control), 4 and 8 µg/mL of FLC addition for 6 h (Fig. 5). Interestingly, the change of mass spectral intensity at range 3376–3382 m/z (major peak) is significantly related to the FLC susceptibility of S, SDD and R reference strains. Three patterns were observed: compared to 0 µg/mL of FLC, 1)the S strain showed a higher peak with 4 and 8 µg/mL; 2) the SDD strain showed a lower peak at 4 µg/mL and a higher peak at 8 µg/mL; and 3) the R strain showed a slight decrease or no change at 4 and 8 µg/mL. After observing the major peak of all 27 clinical strains, their results showed a similar pattern to the S reference strain (data not shown), that were in line with FLC MIC recognized as susceptible (MIC range 0.25-2 µg/mL) and no change in *CDR1* and *CDR2* overexpression.

## Discussion

Of all *C. albicans* strains without FLC addition in this study, only *CDR1* and *CDR2* genes expression are related to FLC SDD (ΔMYA4440) and R strains (ATCC96901). These results confirm the resistance mechanism via increased efflux of the drug from cells by ABC transporters. The R strain has a higher expression of *CDR1* and *CDR2* than the SDD strain. Although only two samples -- consisting of one spontaneous mutant SDD strain and one R reference strain -- are available in this study, the results are in line with previous reports that high expression levels of *CDR* efflux genes is a major mechanism for fluconazole resistance in *C. albicans* [6]. *MDR1* expression in our study was not significantly different among S, SDD and R strains (*p* -value > 0.0001). This may support a previous study which revealed that in planktonic cells *CDR* displays higher expression level after 24 h incubation, while *MDR1* expression is even higher after 48 h [10]. Contrary to our study which revealed that only *CDR1* overexpressed in SDD and R strains after 24 h incubation with FLC, a previous study observed that FLC could induce the expression of *CDR1, CDR2* and *MDR1* [10]. Expression of *CDR1* was controlled five cis-acting regulatory elements including one BEE (basal expression element), one DRE (drug-sensitive element), two SRE (sterol regulatory element) and one NRE (negative regulatory element) while *CDR2* expression was controlled by only DRE. Among these different elements, only DRE is engaged in essential high expression and up-regulation of *CDR1* and *CDR2* to fluconazole efflux [11, 12]. Contrarily, *MDR1* gene does not possess the DRE element, but also *MDR1* promoter does not directly react towards fluconazole [13]. For *ERG11* expression, all strains showed an increase after incubation with 8 µg/mL FLC for 24 h. This supported the previous report that the expression of *ERG11* increased after antifungal treatment in susceptible and non-susceptible to azole isolates [14]. The most interesting comes to *ERG11* gene, the overexpression of which is only partially responsible for the R status. In most cases SNP mutations, which lead to amino acid substitutions that makes the whole protein unable to bind the drug [15]. Additionally, the potential usage of the expression of upregulation transcription factors including *TAC1*, *MRR1*, and *UPC2* in clinical fluconazole-resistant *C. albicans* isolates, which is directly responsible for binding with *CDR*, *MDR1* and *ERG11* promoters, respectively [16]. Notwithstanding, the susceptibility or resistance of *C. albicans* towards fluconazole dose not only originate in down- or over-expression of *CDR1, CDR2, MDR1* or *ERG11* genes. In fact, this might be true to some extent, however, in *C. albicans* cells, it is not a simple dependence. In case of *CDR* genes, the higher amount of mRNA potentially leads to higher protein levels and higher azole efflux. However, *CDR* genes might harbour SNP (single nucleotide polymorphism) mutations, which cannot be sensu stricto interpreted as an “overexpression” [5, 15]. On the other hand, the lack of ergosterol or abnormal sterol deposition in plasma membrane (as an effect of azole treatment) might lead to dysfunction of membrane proteins, including ABC transporters, and affected the mislocalization of the Cdr proteins from plasma membrane to the inside of the cell, which in turn results in the protein inactivity (despite their higher levels) [17]. Concerning our attempt to determine FLC susceptibility to *C. albicans* with MALDI-TOF MS, we found only one spectrum at position 3376–3382 m/z that was linked with FLC resistance of *C. albicans* after incubation with 4 and 8 µg/mL FLC for 6 h. Indeed, by allowing a peak position’s tolerance of ± 3 m/z we were able to overcome the small spectral variations and so could assess as *C. albicans* housekeeping peaks [18]. The drug concentration and incubation time period seem to be major factors for each *Candida* species to observe a major peak for differentiating S strain from R strain. In view of the study by Paul, both the resistant and susceptible *C. tropicalis* isolates showed spectral changes after 4 h when challenged with 128 µg/mL and 1 µg/mL FLC, respectively [19]. In our study, the major mass spectral intensity change was clearly seen after 6 h incubation with FLC. As reported by Vatanshenassan using MBT ASTRA prototype software, the discrimination between resistant and susceptible strains was accurately detected in 6 h [20]. Furthermore, a previous report explained that prolonged exposure to antifungal drug in some isolates allowed an increasing probability of correctly differentiating between S and R strains [8]. Together with the condition of drug concentration and incubation time period, the method processed in each study could support the identification of FLC resistant *C. albicans* strains as well as finding different major mass spectra. For example, in the study of Marinach [21], MPCC determination was used to identify FLC resistant *C. albicans* strains with MALDI-TOF MS. Their study concluded that the most suitable FLC concentrations were 2 and 4 µg/mL and the significant spectral intensity range was 5,800–7,600 m/z to detect FLC resistance. Six spectra in this range could detect FLC resistance of different strains. Many studies have used different procedures including MPCC method [19, 21], composite correlation index (CCI) values approach [8, 22, 23], matrix-assisted laser desorption ionization Biotyper antibiotic susceptibility test rapid assay (MBT-ASTRA) [1, 9] and machine-learning algorithms with the most robust pipeline of analysis [18]. Now, we disclose a specific mass spectrum for analyzing FLC S, SDD and R strain for *C. albicans* with their resistance related to *CDR* expression efflux pump. Our results, which followed the same protocol, come from more than 27 *C. albicans* strains from clinical samples at various sites of 27 patients which were investigated to find the major peak type by MALDI-TOF MS and FLC MIC by BMD. All strains presented a novel mass spectral intensity at range 3376–3382 m/z (major peak) and the major peak type was observed as S type and interpreted MIC level as susceptible (data not shown). Unfortunately, we could not find FLC SDD or R *C. albicans* strain from blood cultures of candidemic patients in the University Hospitals during our study. Moreover, we followed the protocol for determining in 48 *C. tropicalis* strains including FLC S, SDD and R strains from blood cultures of 48 patients. Unsuccessfully, the major peak type could not be used for differentiating *C. tropicalis* (data not shown). For this reason, this major peak type is specific for determining FLC susceptibility testing of *C. albicans*.

## Conclusions

Overall, we discovered a novel mass spectral intensity at range 3376–3382 m/z (major peak) related to the FLC susceptibility of S, SDD and R strains specific for *C. albicans*. The FLC resistance mechanisms of *C. albicans* associated with *CDR1* and *CDR2* expression (via increased efflux of the drug from cells by ABC transporters) may possibly effect the change of mass spectral intensity at range 3376–3382 m/z. The results demonstrate that the MALDI-TOF MS may be used to simultaneously classify *Candida* species and predict FLC resistant *C. albicans* strains associated with *CDR1* and *CDR2* overexpression. However, the limitations of our study include the small number of clinical *C. albicans* strains, the shortage of SDD and R clinical *C. albicans* strains, and the unproven protein spectrum at position 3376–3382 m/z. In addition, several molecular mechanisms of fluconazole resistance except the overexpression have not been considered such as insertions, deletions and point mutations. Therefore, further studies are essential to clarify the methodology and improve the reliability of this assay for routine diagnosis.

## Methods

### Yeast samples used in this study

The *C. albicans* samples in this study were 15 strains isolated from abdomen (1 strains), blood (2 strains), wound/pus (4 strains), sputum (2 strains), stool (1 strain), and urine (5 strains) in the Microbiology Laboratory Unit, Department of Central Laboratory and Blood Bank, Faculty of Medicine, Vajira Hospital, Navamindradhiraj University, Bangkok, Thailand between April 2015 and December 2016, and 12 strains obtained from positive blood culture in the Microbiology Laboratory, Thammasat University Hospital from January 2012 to April 2015. Four ATCC standard strains were used as the control strains including two susceptible strains (*C. albicans* MYA2876 and *C. albicans* ATCC90028), one strain for spontaneous mutation in SDD MIC level (*C. albicans* MYA4440), and one resistant strain (*C. albicans* ATCC96901). *C. parapsilosis*ATCC22019 and *C. krusei*ATCC6258 were used as quality control strains for susceptibility testing.

For this study, we obtained all yeast strains from routine laboratory collection where their data were done namelessly. Therefore, we did not use any additional data other than those gained from laboratory. Human ethical committees decided to exempt, constituted in accordance with the International Conference on Harmonization-Good Clinical Practice (ICH-GCP).

### SensititreYeastOne antifungal susceptibility testing

Twelve strains collected from Thammasat Hospital were subculturedon Sabouraud Dextrose Agar (SDA) and incubated at 35 °C overnight. The procedure used SensititreYeastOne (SYO) according to the manufacturer’s instructions (Thermo Fisher Scientific, Cleveland, OH, USA)for measuring MIC of nine drugs – namely, fluconazole, anidulafungin, amphotericin B, micafungin, caspofungin, 5-Flucytosine, posaconazole, voriconazole and itraconazole. The SYO plate was incubated at 35 °C overnight and MIC endpoints were read after 24 h of incubation by the color change from blue (negative, indicating no growth) to magenta (positive, indicating growth) [24].

### CLSI broth microdilution method (CLSI BMD)

Reference BMD testing was performed exactly as outlined in CLSI document M27-4th ed [25], with a final inoculum concentration of 0.5 × 10^3^ to 2.5 × 10^3^ cells/mL and RPMI 1640 medium with 0.2% glucose, and incubation at 35 °C. MIC values were determined by visually observing the presence or absence of growth after 24 h (*Candida* and other yeast) and 72 h (*C. neoformans)* as the lowest concentration of drug that produced a prominent decrease in turbidity (ca. 50% growth reduction) relative to that of the drug-free control.

### Susceptibility breakpoints

The MIC (µg/mL) interpretations follow the CLSI breakpoints for fluconazole (MIC ≤ 2 µg/mL, Susceptible; MIC = 4 µg/mL, Susceptible-dose dependent; MIC ≥ 8 µg/mL, Resistant). Interpretation of susceptibility was performed by applying the CBPs defined by CLSI [26]. In the CBPs absence, isolates were defined as having a wild-type or a non-wild-type drug susceptibility phenotype including amphotericin, 5-fucytosine, itraconazole and posaconazole according to the epidemiological cutoff values determined by SYO [24, 27].

### Selection of spontaneous mutants

*C. albicans* MYA4440 isolates (MIC = 0.5 µg/mL) and G950 isolates (MIC = 2 µg/mL) were subcultured and incubated at 35 °C overnight. The individual colony from each strain was used to start cultures in RPMI and grown overnight. One hundred microliters of overnight cultures (5 × 10^8^ to 1 × 10^9^ CFU/mL) were streaked onto SDA plates with (1, 1.5, 2, and 4 µg/mL) or without fluconazole and incubated at 35 °C for 2 days. MIC was checked and resubcultured onto a fresh-passage until the MIC was 4 µg/mL. The spontaneous mutation frequency rate was calculated as the ratio of viable colonies growing on drug-containing plates over the starting inoculum. Mutant resistance phenotypes were confirmed by subculturing on SDA plates containing an amount of drug equivalent to that used for initial selection. Mutant strains were selected and evaluated by MIC and qRT-PCR.

### RNA extraction, cDNA synthesis and quantitative real-time PCR (qRT-PCR)

Each *C. albicans* isolate was suspended in 5 mL of yeast nitrogen base (YNB) medium supplemented with 50 mM glucose and incubated at 37 °C in a shaker at 75 rpm overnight to prepare each of the starters. After that, 500 µL of the starter were transferred into a flask of 50 mL containing fresh medium and incubated at 37 °C in a shaker at 75 rpm until OD_600_ = 1.0. Total RNA was extracted from the growth medium (at 0 h without fluconazole and after 24 h with 8 µg/mL of fluconazole addition) at mid-exponential (log) phase using the preparation of yeast RNA by extraction with hot acidic phenol. The concentration of RNA was measured using a NanoDrop2000C spectrophotometer (Thermo Scientific). The RNA purity and integrity was evaluated by the ratio of absorbance 260 and 280 nm. Run gel electrophoresis was performed to verify that the RNA was intact. Total RNA was treated with TURBO DNA-*free*™ Kit (Invitrogen) according to the manufacturer’s instructions (RNA sample > 200 µg nucleic acid per mL). cDNA was synthesized by reverse transcription from 1 µg of total RNA using the iScript™ Reverse Transcription Supermix for RT-qPCR (Biorad) according to the manufacturer’s instructions. The reaction protocols for cDNA synthesis were composed of a priming step at 25 °C for 5 min, a reverse transcription step at 46 °C for 20 min, and an RT inactivation step at 95 °C for 1 min. The mRNA expression level was measured using quantitative real-time RT-PCR (qRT-PCR) following Watamoto’s protocol [28]. The sequence primers (macrogen) of the genes *CDR1, CDR2, MDR1, ERG11* [28], and *PMA1* [29] in this study are listed in Table 3. qRT-PCR was performed in duplicate using iTaq™ Universal SYBR® Green Supermix (Biorad). Ten microlitres of PCR mix (5 µLiTaq™ Universal SYBR® Green Supermix, 1 µL primer mix, 0.5 µL cDNA and 3.5 µL DEPC water) was used for each gene and qRT-PCR was performed using the following cycling conditions: 95 °C for 5 min, followed by 40 cycles of 95 °C for 15 s and 60 °C for 30 s. Fluorescence intensities were quantified using Bio-Rad® CFX96™ (Biorad). The relative quantities of the target genes (*CDR1, CDR2, MDR1* and *ERG11*) were normalized against *PMA1* housekeeping gene expression (plasma membrane ATPase pump). The analyses of genes expressions were performed using the comparative 2^(−ΔΔCT)^ method of relative quantification. The mRNA expression level of each target gene was analyzed. All results were presented by mean ± SD. Statistical analyses were performed using GraphPad Prism 5.0 software. Results were compared using one way ANOVA followed by Bonferroni’s post test; the results were considered statistically significant when the *p* -value was < 0.0001.

**Table 3:** Gene-specific primers for qRT-PCR [22]

Target gene	Sequence (5’◊3’)	Amplicon size (bp)	Reference
*PMA1*	F: 5’-TTGCTTATGATAATGCTCCATACGA-3’ R: 5’-TACCCCACAATCTTGGCAAGT-3’	-	[23]
*CDR1*	F: 5’-GTACTATCCATCAACCATCAGCACTT-3’ R: 5’-GCCGTTCTTCCACCTTTTTGTA-3’	79	[22]
*CDR2*	F: 5’-TGCTGAACCGACAGACTCAGTT-3’ R: 5’-AAGAGATTGCCAATTGTCCCATA-3’	104	[22]
*MDR1*	F: 5’-TCAGTCCGATGTCAGAAAATGC-3’ R: 5’-GCAGTGGGAATTTGTAGTATGACAA-3’	91	[22]
*ERG11*	F: 5’-GGTGGTGATTTGAATGATTTGACTTAT-3’ R: 5’-GGCATATGCATTCTAAGAGTTTCCT-3’	92	[22]

*F* Forward, *R* Reverse

### Detection of mass spectral analysis using MALDI-TOF MS

Fresh-isolated colonies on SDA were cultured in RPMI 1640 medium, adjusted turbidity with 0.5 McFarland Standard, and incubated at 37 °C overnight. Two milliliters (mL) of suspension were added into RPMI 1640 medium with different fluconazole concentrations: 4 and8 µg/mL, and without fluconazole as a negative control. The suspension was incubated at 37 °C and harvested at 6 h intervals, centrifuged and the pellets and the supernatant separated. The pellets were extracted with formic acid following Bruker Daltonics’ recommended protocol for analysis of *C. albicans* protein spectrum. Briefly, 300 µL of HPLC grade water and 700 µL of ethanol were added, mixed with a vortex for 1 min, and centrifuged at 13,000 rpm for3 min. The solution was discarded after centrifugation. After drying the pellets, 70% formic acid was added to cover the pellets, then silica beads were added into the solution before mixing with vortex for 2 min. Acetonitrile was added in equal volume of 70% formic acid (ratio 1:1). The mixer was centrifuged at 13,000 rpm for 3 min. One microliter of supernatant was spotted on the MALDI target plate and dried at room temperature (RT). One microliter of 2.5% TFA of α-Cyano-4-hydroxycinnamic acid (HCCA) matrix was covered and dried at RT again. The sample was analyzed by MALDI-TOF MS (Bruker Daltonics, Bremen, Germany). A drop of the supernatant used for fluconazole spectrum analysis was placed onto a plate and dried, covered with0.1% TFA of HCCA matrix, dried at RT, and analyzed by Bruker AutoflexSpeed MALDI-TOF MS. The bacterial test standard (BTS), the ribosomal proteins of *Escherichia coli* strain DH5alpha, was used as a calibrator. The spectrum was measured by flexControl 3.3 software [19, 30] using linear mode (2,000–20,000 m/z) for *C. albicans* spectrum and using reflector mode (0–3,500 m/z) for fluconazole spectrum. Finally, the data was processed and compared using flexAnalysis.

## Data Availability

All the data required are included in the manuscript.

## Abbreviations

*C. albicans*: *Candida albicans*
MALDI-TOF MS: Matrix-assisted laser desorption ionization-time of flight mass spectrometry
Real-time PCR: Real-time polymerase chain reaction
*CDR*: *Candida *drug resistance
*MDR*: Multidrug resistance
*ERG*: Ergosterol biosynthesis
FLC: Fluconazole
S: Susceptible
R: Resistant
SDD: Susceptible-dose dependent
mRNA: Messenger ribonucleic acid
MIC: Minimal inhibitory concentration
ABC transporters: ATP-binding cassette transporters
MFS: Major facilitator superfamily
BMD: Broth microdilution
AFST: Antifungal susceptibility test
CLSI: Clinical and Laboratory Standards Institute
EUCAST: European Committee on Antibiotic Susceptibility Testing
SYO: SensititreYeastOne
CBPs: Clinical breakpoints
ECVs: Epidemiological cutoff values
MPCC: Minimal profile changing concentration
CCI: Composite correlation index
BEE element: Basal expression element
DRE: Drug response element
SRE: Sterol regulatory element
NRE: Negative regulatory element
SNP mutations: Single nucleotide polymorphism mutations
MBT-ASTRA: Matrix-assisted laser desorption ionization Biotyper antibiotic susceptibility test rapid assay
SDA: Sabouraud Dextrose Agar
RPMI 1640 medium: Roswell Park Memorial Institute 1640 culture medium
cDNA: Complementary deoxyribonucleic acid
qRT-PCR: Quantitative real-time polymerase chain reaction
YNB: Yeast nitrogen base
OD: Optical density
RT-qPCR: Quantitative reverse transcription polymerase chain reaction
RT inactivation: Reverse transcriptase inactivation
*PMA1*: Plasma membrane ATPase pump
DEPC: Diethyl pyrocarbonate
HPLC: High performance liquid chromatography
RT: Room temperature
TFA: Trifluoroacetic acid
HCCA: α-Cyano-4-hydroxycinnamic acid
BTS: Bacterial test standard
